# MicroRNA–mRNA Regulatory Network Associated with Cognitive Impairment in Multiple Sclerosis

**DOI:** 10.1007/s12035-026-05893-x

**Published:** 2026-04-30

**Authors:** Bruna De Felice, Federica Farinella, Giuseppe Romano, Concetta Montanino, Simona Bonavita, Deborah Archetto, Elisabetta Maida, Simona Raimo, Giacomo Lus, Maria Cropano, Cinzia Coppola, Elisabetta Signoriello

**Affiliations:** 1 Department of Environmental, Biological and Pharmaceutical Sciences and Technologies, University of Campania Luigi Vanvitelli, Caserta, Italy; 2 Department of Advanced Medical and Surgical Sciences, University of Campania “Luigi Vanvitelli”, Via Vivaldi 43, 81100 Caserta, Italy; 3 Department of Medical and Surgical Sciences, “Magna Graecia” University of Catanzaro, Catanzaro, Italy; 4 Department of Health Sciences, “Magna Graecia” University of Catanzaro, Catanzaro, Italy

**Keywords:** Multiple sclerosis, Cognitive impairment, MicroRNAs, MRNAs

## Abstract

Cognitive impairment (CI) is a frequent and disabling manifestation of multiple sclerosis (MS), yet its molecular underpinnings remain poorly understood. This study aimed to identify microRNA (miRNA) signatures and related gene expression changes associated with CI in MS. Forty-six people with MS underwent clinical, radiological, and cognitive assessment. Peripheral blood mononuclear cells were collected for miRNA profiling using the miRCURY LNA miRNA Focus PCR Panel and validated through RT-qPCR. Experimentally validated miRNA target genes were retrieved using miRWalk with miRTarBase filtering. Target networks were constructed in Cytoscape, followed by protein–protein interaction analysis using STRING and functional enrichment analysis with database for annotation, visualization, and integrated discovery (DAVID) (https://davidbioinformatics.nih.gov/). Selected target genes were further evaluated by gene expression analysis. The results showed that three miRNAs (i.e., miR-146a-5p, miR-let-7a-5p, and miR-21-5p) were significantly dysregulated in patients with MS with CI compared to those with preserved cognition. Gene expression analysis identified differential regulations of IL-1B, IL-6, SLC16A10, and NEFL, supporting the involvement of inflammatory and neurodegenerative pathways. Correlation analyses indicated specific miRNA–mRNA relationships underlying these alterations. These findings suggest that the combined dysregulation of miR-146a-5p and miR-21-5p, together with their target genes, constitutes a molecular signature associated with CI in MS. This profile could contribute to the development of miRNA-based biomarkers for early detection and monitoring of cognitive dysfunction in MS.

## Introduction

Multiple sclerosis (MS) is a chronic, immune-mediated, demyelinating, neurodegenerative disorder that affects the central nervous system (CNS). MS leads to a wide range of physical, cognitive, and emotional impairments, imposing a substantial burden on patients, their families, and healthcare systems [1–3]. MS commonly affects multiple CNS functions, often presenting with motor or sensory symptoms due to white matter lesions in the brain, spinal cord, or optic nerve. In addition, cognitive impairment (CI) is also a prevalent feature, affecting approximately 34–65% of people with MS, varying by research setting and disease course [4, 5]. It can be detected even in early disease stages, such as radiologically isolated syndrome (RIS), and tends to worsen with disease progression [5]. CI can emerge suddenly during relapses or develop progressively over time. The prevalence of CI varies across disease phenotypes, with estimates of 20–25% in clinically isolated syndrome (CIS) and RIS, 30–45% in relapsing-remitting MS (RRMS), and 50–75% in secondary progressive MS (SPMS), while rates in primary progressive MS (PPMS) remain highly variable [6]. Despite these broad estimates, CI profiles can differ significantly among individuals with the same disease phenotype. Nevertheless, the most affected cognitive domains typically include processing speed, working memory, visuospatial skills, and executive function [7, 8]. CI in MS results from damage to both white and gray matter [9–12]. However, white matter lesions alone do not fully account for cognitive decline, while atrophy of gray matter, particularly in areas like the thalamus, hippocampus, and cortex, has been strongly linked to cognitive deterioration. This increased involvement of gray and deep matter may reflect the underlying substrate of smouldering inflammation, a phenomenon that is gaining more recognition in MS [6]. Accordingly, CI may represent a sensitive clinical correlation of diffuse neurodegenerative and inflammatory mechanisms occurring early and progressively throughout the course of the disease [13]. Despite its high prevalence and clinical relevance, the molecular mechanisms underlying CI in MS remain poorly understood, and reliable biomarkers for early detection, prognosis, and patient stratification are still lacking [14–16]. MicroRNAs are small single-stranded RNAs typically about 19–24 nucleotides long which regulate the expression of genes and are responsible for a variety of diverse biological processes, including differentiation and proliferation of cells, their metabolism, inflammation, and apoptosis [17]. MiRs control translation of proteins responsible for myelin repair, gliogenesis, and neurogenesis, and participate in the control of neuronal identity, differentiation, and proliferation [18]. The dysregulation of certain miRs is responsible for neurodegeneration and autoimmune processes [19]. Several miRNAs are known to be dysregulated in MS, and those overlapping with miRNA alterations observed in neurodegenerative dementias may play a role in the development of CI in MS [20]. On this basis, the present study aimed to identify dysregulated microRNAs and related mRNA changes associated with cognitive impairment in patients with MS, comparing individuals with cognitive impairment (MS + CI) and those with preserved cognition (MS-CI).

## Materials and Methods

### Recruitment of Participants

This was a prospective observational study conducted at the MS Center, II Neurology Department of the AOU Luigi Vanvitelli. We enrolled individuals with suspected demyelinating disease who were referred to our center, between February 2021 and May 2023. All participants underwent a comprehensive diagnostic work-up, including clinical evaluation, radiological and neuropsychological assessment, and lumbar puncture, to confirm a diagnosis of MS according to the McDonald 2017 criteria [21]. For these participants, we collected the following data: clinical and demographic information, including sex, age, and Expanded Disability Status Scale Score [22]; radiological data, such as the number of gadolinium-enhancing lesions, spinal cord lesions, and brain lesion load, defined as high if more than nine brain lesions were present. All participants provided informed consent, and the study followed the ethical guidelines established by the Ethics Committee of the University of Campania (Prot. 12,478/20). Table 1 summarizes cohort details and inclusion/exclusion criteria.

**Table 1:** Clinical and demographic characteristics of the MS patients

	MS (*n* = 46)	MS + CI (*n* = 23)	MS-CI (*n* = 23)	*p*-value
Age, mean ± SD	36.8 ± 12	38.6 ± 11.1	36.7 ± 12.1	0.58
Female sex, *n* (%)	29 (63%)	14 (31.8%)	14 (31.8%)	-
Time to onset, mean ± SD	1.76 ± 3.18	2.33 ± 3.56	1.31 ± 2.88	0.307
EDSS, mean, SD	1.78 ± 1.39	1.86 ± 1.42	1.75 ± 1.45	0.632
Spinal cord lesions, mean, SD	1.64 ± 1.85	1.85 ± 1.13	1.6 ± 1.57	0.944
High encephalic load, *n* (%)	21 (48.8%)	12 (29.3%)	7 (17.1%)	0.155

### Neuropsychological Assessment

On the day of the lumbar puncture, cognitive functioning was evaluated using the Brief International Cognitive Assessment for Multiple Sclerosis (BICAMS) [23, 24], a brief neuropsychological battery specifically developed for patients with MS. It includes the following: (i) the symbol digit modalities test (SDMT), assessing information processing speed and attention abilities; (ii) the California verbal learning test-second edition (CVLT-II), which evaluates verbal learning and memory abilities; and (iii) the brief visuospatial memory test-revised (BVMT-R), assessing visuospatial learning and memory abilities. For each subtest, raw scores were adjusted for age, sex, and education according to normative data for the Italian population [7]. CI was defined as a performance below the normative cut-off on at least one BICAMS subtest, consistent with previous studies in MS [4, 25].

### Blood Collection

From each participant, a venous blood sample was drawn in connection with the administration of the BICAMS test. The sample-handling procedures were carried out in accordance with the Helsinki Declaration. Peripheral blood mononuclear cells (PBMC) were isolated from EDTA blood using one RBC and one PBS wash. About 1 × 10^7^ reconstituted cells were stored in Qiazol® (Qiagen, Hilden, Germany) at −80 °C.

### RNA Extraction

Following the manufacturer’s instructions, total RNA was extracted using the Qiazol® technique plus an optional DNase digestion step. With the aid of a NanoDrop spectrophotometer (NanoDrop technologies, USA), the quality and amount of RNA were assessed.

### RNA Reverse Transcriptase

Using the Takara-Bio PrimeScript RT Reagent Kit with gDNA for genes, 1 µg of total RNA was reverse-transcribed for gene expression analysis. Additionally, 1 µg of total RNA was utilized with the Takara-Bio Mir-X™ miRNA First-Strand Synthesis Kit to acquire complementary microRNAs.

### MicroRNAs Profiling

miRCURY LNA miRNA Focus PCR Panel (Qiagen) examining 84 microRNAs was used to profile the miRNome. The ABI real-time apparatus (ABI StepOnePlus, USA) was utilized to perform qPCR array reactions. To conduct the reaction, 450 µL of RNase-free water, 500 µL of (2×) Fast SYBR-Green PCR Mix (Qiagen), and 5 µL of undiluted cDNA were combined. The amount of the sample related to each MS patient examined using BICAMS test consisted of 1 µg of RNA. Internal controls SNORD44, SNORD38B, SNORD49A, and U6 snRNA were used to calculate ∆Ct.

### Validation of MicroRNAs Expression in Blood by Real-Time Quantitative PCR (RT-qPCR)

RT-PCR quantification of differentially expressed microRNAs, in duplicate for each sample using microRNA assays (Applied Biosystems Inc., Foster City, CA, USA) was conducted, and melting curve analysis was used to assess amplification profiles and exclude evident nonspecific products as part of the routine quality control procedure. The ABI StepOnePlus machine (Applied Biosystems, Foster City, CA, USA) was used for RT-PCR. For reactions, a mixture (20 µL) containing 2 µL cDNA template, 10 µL (2 ×) Fast SYBR-Green PCR Mix (Qiagen), and 1 µL each of sense and antisense primers was used. Forward primer sequences of target microRNAs are reported in Table 2. MicroRNAs expression level, using U6 as the internal control, was obtained using the 2^−ΔΔCt^ method. Prior to the validation phase, candidate endogenous controls were preliminarily assessed in our sample set, and U6 showed the most stable performance across groups; it was therefore selected as the endogenous control for RT-qPCR validation.

**Table 2:** Primer sequences used for RT-qPCR of miRNAs

miRNAs name	Primer sequence
miR-21a-5p	5′-ACA CTC CAG CTG GGT AGC TTA TCA GAC TGA-3′
miR-146a-5p	5-TGA GAA CTG AAT TCC ATG GGT T-3′
miR-let-7a-5p	5′-ACA CTC CAG CTG GGT GAG GTA GTA GTT TGT-3′
Universal real-time reverse primer	5′-GTG TCG TGG AGT CGG CAA TTC-3′

### Quantitative Real‑Time PCR (RT‑qPCR) Validation of Predicted Genes

We performed RT-qPCR analysis to verify the expression of genes involved in inflammation and neurodegeneration, in peripheral blood of patients with MS. Briefly, total RNA was extracted using Qiazol® reagent (Qiagen, Hilden, Germany); after assessment of RNA quality and concentration, reverse transcription and RT-qPCR were performed using the Takara Prime-Script RT Master Mix (RR036A) and Fast SYBR-Green PCR Mix (Qiagen, Hilden, Germany), respectively. Primer sequences of target genes were reported in Table 3. RT-qPCR was performed in technical triplicate per target gene. Relative transcript abundance was determined by using the 2^−ΔΔCt^ method. Prior to RT-qPCR analysis, candidate housekeeping genes were preliminarily evaluated in our sample set, including GAPDH and β-actin. Based on their performance across the study groups, β-actin was selected as the endogenous control for normalization of target gene expression.

**Table 3:** Primer sequences of target genes

Gene name	Primer sequence
IL-1B	forward 5′-TGA GGG TAT CGC AGA GAA CGG A-3′ reverse 5′-CGG TCA CTT ATC CTG TGG CTG G-3′
IL-6	forward 5′-CTG TTC CTC ACA GCC ATC GAC A-3′ reverse 5′-TGG CTA TGG TCC ACA TCA GGC A-3′
SLC16A10	forward 5′-GAG CAG CCA AGC TGA AGA GAA C-3′ reverse 5′-GCC ATT TCT TAG AGT TCA GGC ATG −3′
NEFL	forward 5′- CCC CAG ACT CCG TCA GTT TCT-3′ reverse 5′-CAT TCT CCG TCT GGT-3′

### Statistical and Bioinformatics Analyses

An unpaired two-tailed *t*-test was performed on normalized ΔCt values to assess differences in gene and microRNA expression between patients with MS with and without cognitive impairment. Relative expression levels were calculated as fold change using the 2^−ΔΔCt^ method. The statistical analysis was carried out using GraphPad Prism 8.4.2. tool.

Experimentally validated target genes of the dysregulated miRNAs (miR-146a-5p, miR-let-7a-5p, and miR-21-5p) were retrieved using the miRWalk platform (http://mirwalk.umm.uni-heidelberg.de/), restricting the analysis to interactions supported by miRTarBase [26] in order to include only experimentally validated miRNA–mRNA associations and exclude purely computational predictions. Target lists were generated independently for each miRNA and subsequently merged to obtain a comprehensive set of validated interacting genes.

The integrated list of target genes was imported into Cytoscape [27, 28] (version 3.10.2) for network visualization and identification of shared targets among the three miRNAs. Overlapping target genes were identified by network topology analysis within Cytoscape.

To evaluate functional relationships among the validated targets, protein–protein interaction (PPI) analysis was performed using the STRING [29, 30] database (https://string-db.org/), considering known and predicted functional associations derived from experimental evidence, curated databases, co-expression, and text mining. The resulting interaction network was visualized to assess connectivity and hub nodes.

Functional enrichment analysis was conducted using the DAVID [31, 32] online tool (https://david.ncifcrf.gov/) with *Homo sapiens* as background. Enrichment was evaluated across Gene Ontology (GO) categories, biological process, molecular function, and cellular component, as well as KEGG and WikiPathways databases. Significantly enriched terms were identified using a false discovery rate (FDR)-adjusted *p*-value threshold of < 0.01.

## Results

### Clinical Characteristics of the Cohort Stratified by Cognitive Status

Table 1 shows the distribution of clinical and demographic variables in the enrolled cohort, stratified according to cognitive status as defined by BICAMS classification. No statistically significant differences were observed between MS + CI and MS − CI in the main clinical or demographic variables.

The study population included 46 patients with MS, 63% of whom were female. The mean age at disease onset was 36.8 ± 12 years in the overall cohort, while the mean EDSS score was 1.78 ± 1.39. A high encephalic lesion load was observed in 48.8% of the overall population. Of the 46 recruited patients with MS, 50% showed alterations in BICAMS at baseline and were therefore classified as cognitively impaired (MS + CI). Among these, 54.5% had deficits in a single BICAMS subtest, 36.4% in two subtests, and 9.2% in all three subtests. The most frequently affected domains were information processing speed (SDMT; 55%) and visuospatial memory (BVMT-R; 55%), followed by verbal memory (CVLT-II; 45%); percentages are not mutually exclusive, as patients with impairments in two or three subtests are counted under each affected domain.

### MicroRNAs Profiling of Patients with MS Evaluated Through BICAMS Test and RT qPCR Validation

**Fig. 1:**
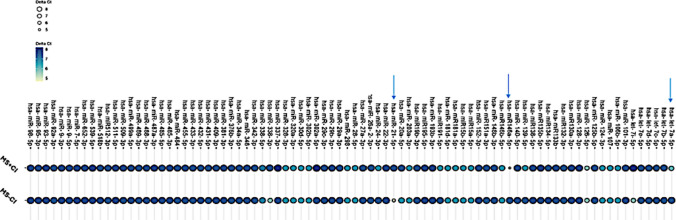
miRCURY LNA miRNA Focus PCR Panel screening analysis. Bubble size and color intensity reflect normalized ΔCt expression levels of each microRNA in patients with MS with and without cognitive impairment. Arrows highlight microRNAs exhibiting statistically significant differential expressions between groups (*p* < 0.05)

**Fig. 2:**
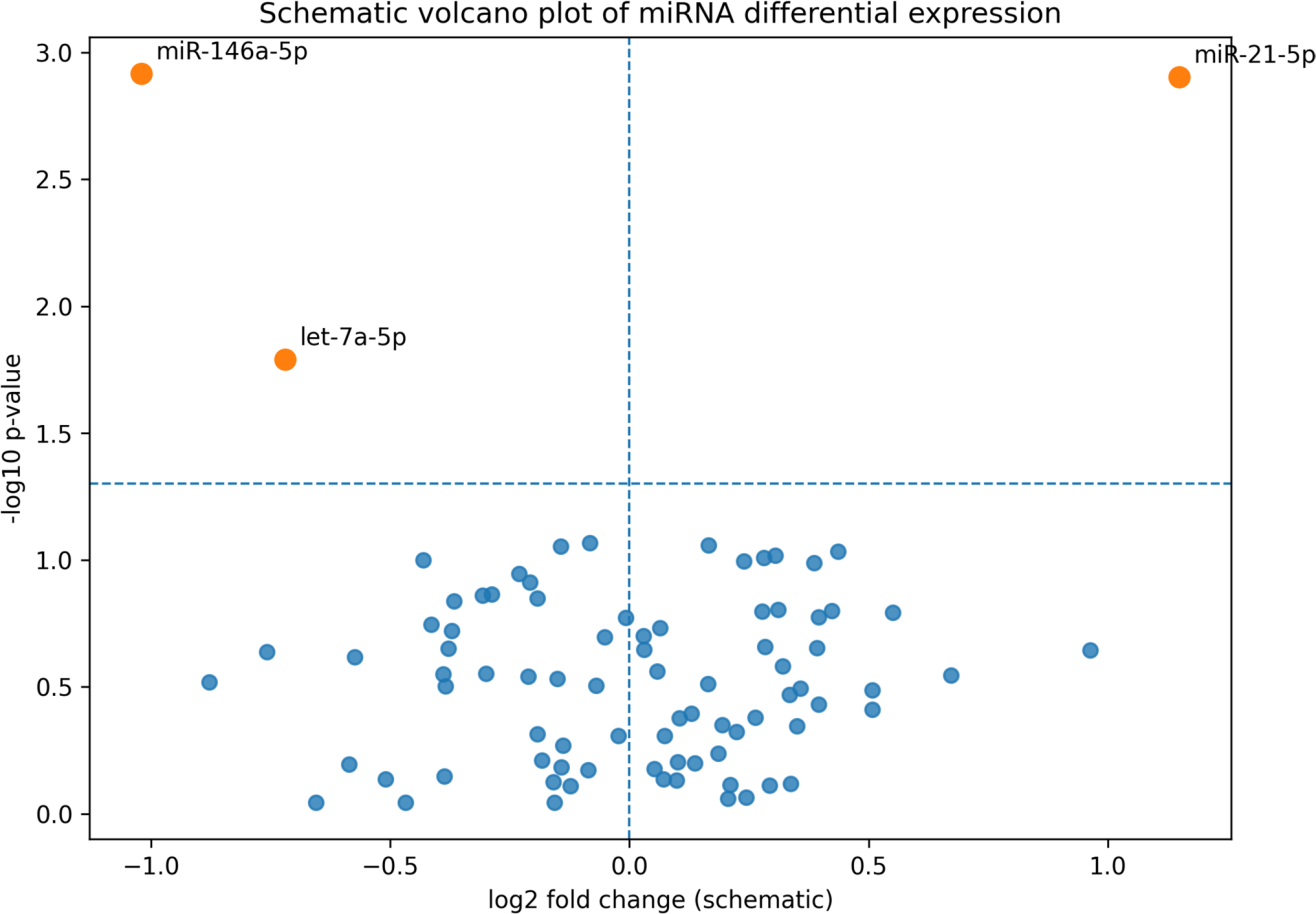
Volcano-style plot. Volcano plot showing differential miRNA expression between MS + CI and MS − CI patients. Significantly dysregulated miRNAs are highlighted in orange

To look for relationships between genetics and the outcomes of a neuropsychological tests battery, 84 microRNAs were examined using miRCURY LNA miRNA Focus PCR Panel (Figs. 1 and 2). All MS samples analyzed exhibited dysregulation in the same microRNAs: miR-let-7a-5p, miR-let-7i-5p, miR-338-3p, miR-26a-2-3p, miR-146a-5p, and miR-21-5p. Of these, only miR-let-7a-5p, miR-146a-5p, and miR-21-5p showed a statistically significant difference between the two groups (Table 4).

**Table 4:** MicroRNAs expression *p*-values in MS + CI and MS − CI groups

MicroRNAs	Direction in MS + CI	Fold change (2^−ΔΔCt^)	*p*-values MS + CI vs MS − CI
mir-let-7a-5p	Downregulated	1.16	0.01619
mir-146a-5p	Downregulated	2.56	0.00121
mir-21-5p	Upregulated	2.51	0.00125

**Fig. 3:**
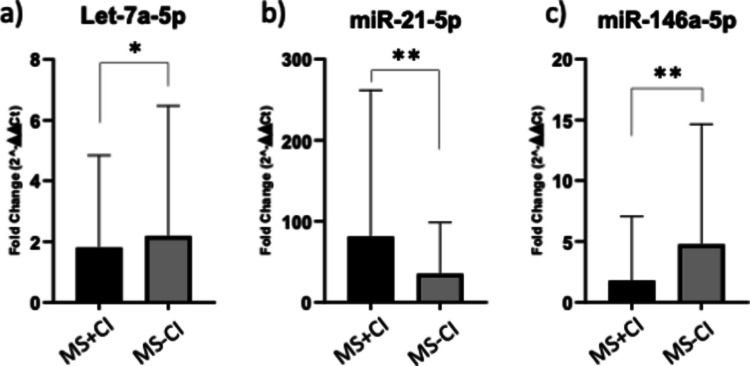
Bar plots showing relative miRNA expression levels expressed as fold change in patients with MS with cognitive impairment (MS + CI) and without cognitive impairment (MS-CI). Error bars indicate mean ± SD. Statistical significance was assessed using an unpaired two-tailed *t*-test. Significance levels are indicated as *p < 0.05 and **p < 0.01

According to microRNA profiling values ∆Ct, MS + CI were found to have lower expression levels of miR-let-7a-5p, and miR-146a-5p, and higher expression levels of miR-21-5p. The expression levels of three dysregulated microRNAs were then confirmed by RT-qPCR analysis. The expression data are shown comprehensively in Fig. 3, which additionally provides a representation of the statistically significant variations in miR-146a-5p, miR-let-7a-5p, and miR-21-5p expression between the two conditions (MS + CI vs MS-CI) also supported by Table 4 data.

### Bioinformatic miRNA-Target Analysis and miRNA–mRNA Interaction Network

After the identification and RT-qPCR validation of the three significantly dysregulated miRNAs (miR-146a-5p, miR-let-7a-5p, and miR-21-5p), a bioinformatic workflow was implemented to explore their potential regulatory landscape.

Experimentally validated target genes were retrieved using the miRWalk platform, restricting the search to interactions confirmed in miRTarBase to ensure high-confidence evidence and avoid purely predictive associations. Target lists were generated independently for each miRNA and subsequently integrated into Cytoscape to construct an interaction network and identify shared regulatory nodes.

**Fig. 4:**
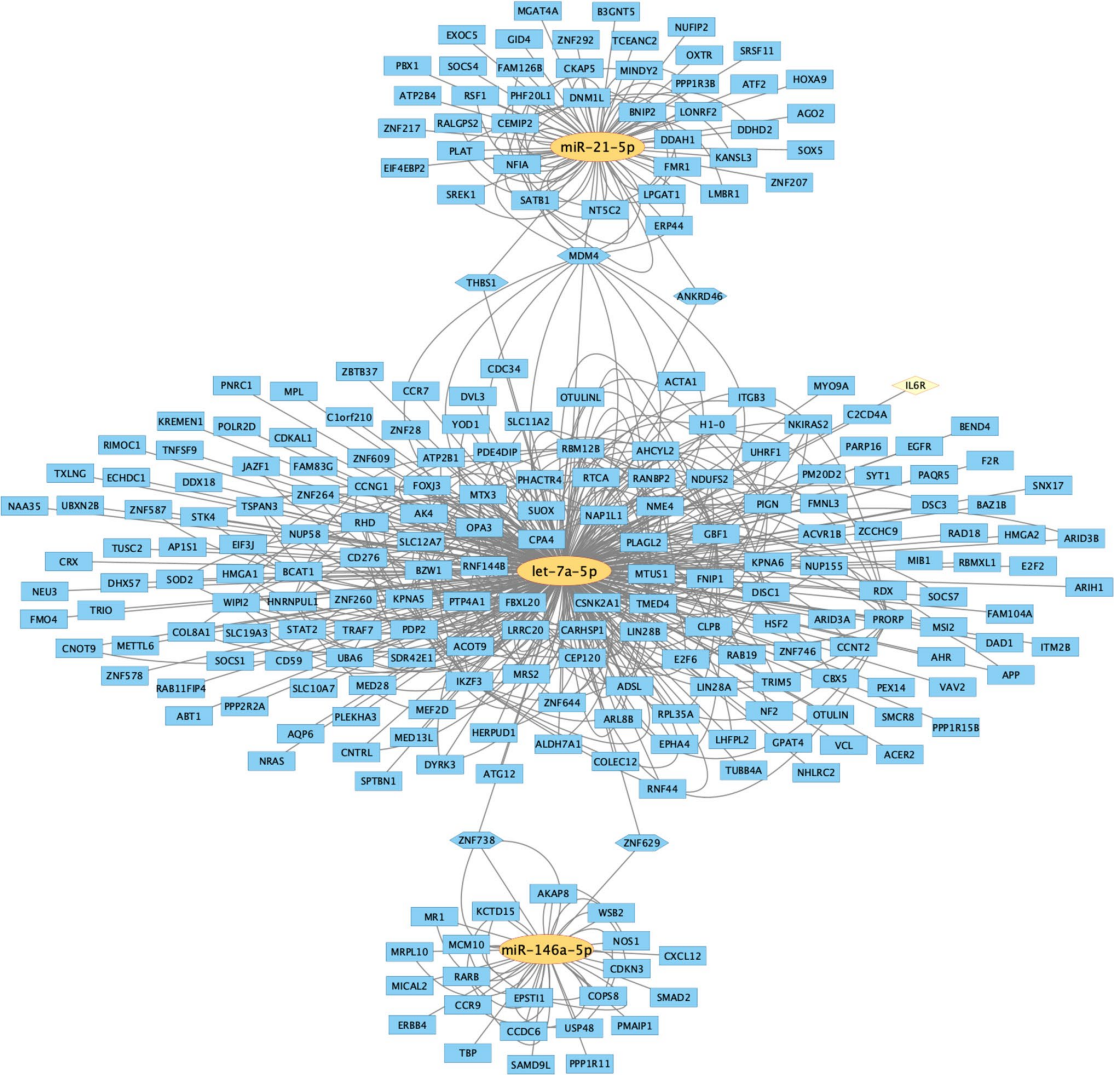
Experimentally validated target genes of miR-146a-5p, miR-let-7a-5p, and miR-21-5p were retrieved from miRTarBase via miRWalk and visualized in Cytoscape. Yellow nodes represent the three dysregulated miRNAs, while blue nodes represent validated target genes. Edges indicate experimentally supported miRNA–mRNA interactions

The network (Fig. 4) highlights a highly interconnected regulatory architecture, with let-7a-5p exhibiting the largest number of validated targets. Shared target genes between miRNAs are positioned at network intersections, including THBS1, MDM4, and ANKRD46 (shared between miR-21-5p and let-7a-5p), and ZNF738 and ZNF629 (shared between let-7a-5p and miR-146a-5p). Notably, IL6R appears as a validated direct target of let-7a-5p, linking the identified miRNA signature to inflammatory signaling pathways. The overall topology suggests overlapping regulatory control across transcriptional, inflammatory, and neurodegenerative processes.

### Bioinformatic Functional Enrichment and Protein–Protein Interaction Network Analysis

**Fig. 5:**
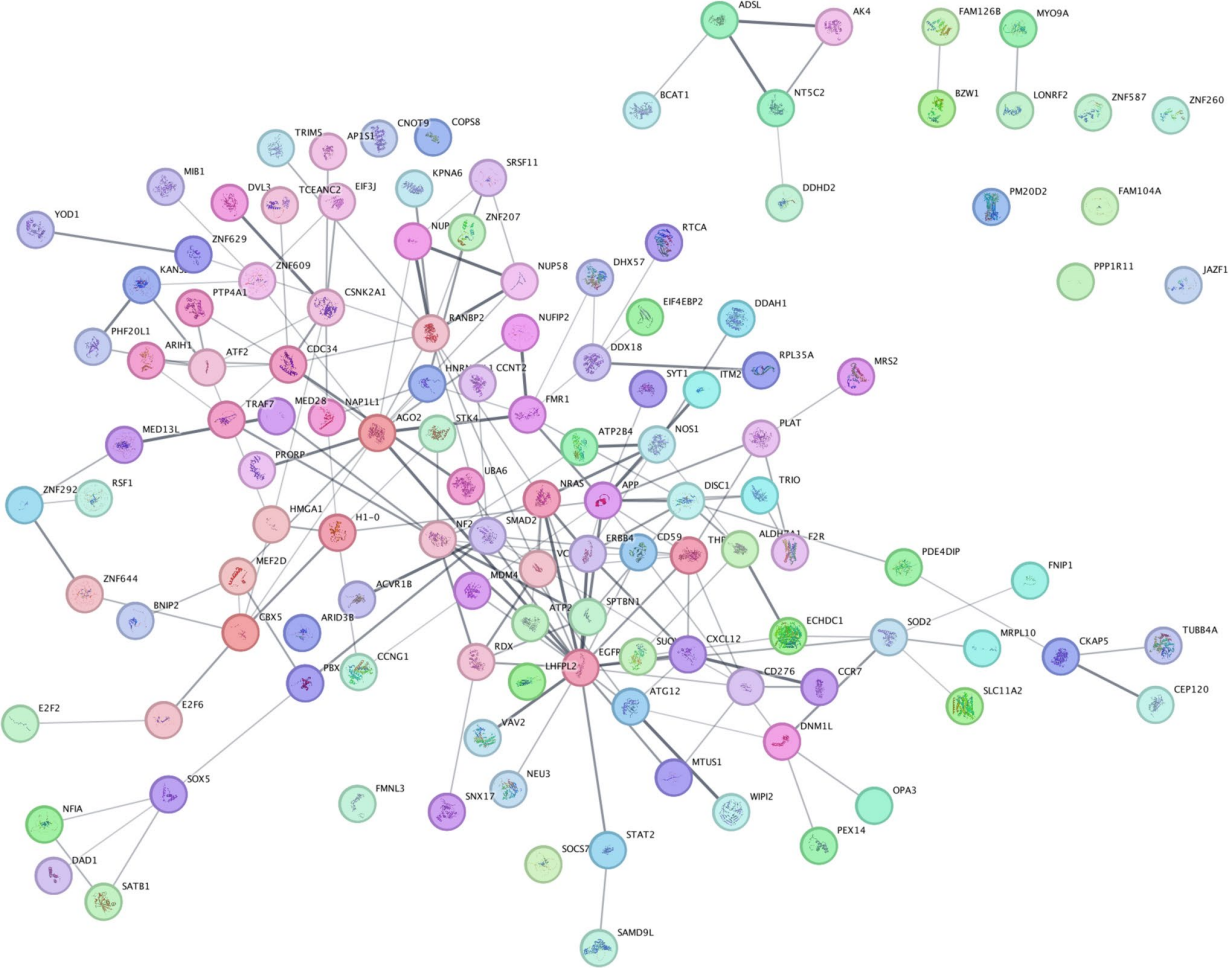
Protein–protein interaction (PPI) network of validated miRNA target genes. The integrated list of experimentally validated targets of miR-146a-5p, miR-let-7a-5p, and miR-21-5p was analyzed using STRING to construct a protein–protein interaction network. Nodes represent proteins and edges represent known or predicted functional associations based on experimental evidence, curated databases, co-expression, and text mining

To further investigate functional relationships among the validated targets, the integrated gene list was analyzed using STRING to generate a protein–protein interaction (PPI) network (Fig. 5). The resulting network displayed a densely interconnected core structure, characterized by several high-degree hub nodes, including APP, EGFR, SMAD2, STAT2, CXCL12, ERBB4, MDM4, and AGO2. Many of these proteins are known to participate in inflammatory signaling, neurodegenerative pathways, and transcriptional regulation, supporting the biological relevance of the identified miRNA–target axis. The presence of modular subclusters within the network further suggests coordinated functional organization rather than random association.

Overall, the convergence of enrichment and PPI analyses indicates that the dysregulated miRNAs identified in patients with MS with cognitive impairment may influence interconnected transcriptional and inflammatory signaling networks, potentially contributing to neurodegenerative processes.

**Fig. 6:**
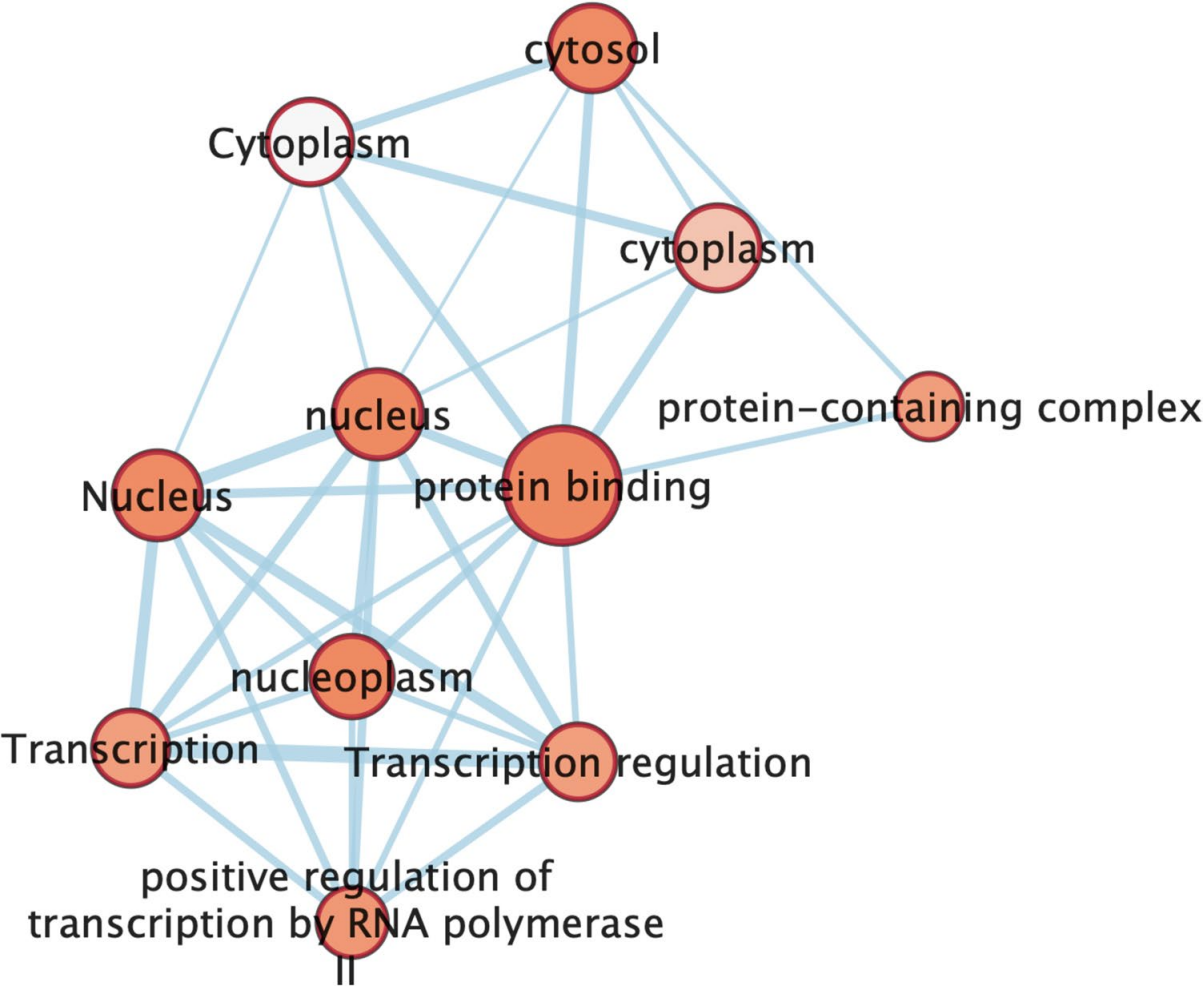
Functional enrichment network of experimentally validated miRNA target genes. Experimentally validated targets of miR-146a-5p, miR-let-7a-5p, and miR-21-5p were retrieved from miRTarBase via miRWalk and subjected to functional enrichment analysis using DAVID. Overrepresented Gene Ontology (GO) terms are displayed as interconnected nodes, with node size reflecting the number of associated genes and edge thickness representing the degree of shared genes between terms

Functional enrichment analysis was then performed using DAVID, interrogating KEGG, Gene Ontology (GO), and WikiPathways databases. Enrichment analysis (Fig. 6) revealed a predominant involvement of nuclear and transcription-related processes. Significantly overrepresented GO terms included nucleus, nucleoplasm, transcription, and regulation of transcription, particularly positive regulation of transcription by RNA polymerase II. The term protein binding emerged as a highly connected central node, indicating extensive interaction capacity among the identified targets. In addition, cytosolic and cytoplasmic components were significantly enriched, suggesting that the regulatory landscape extends beyond nuclear transcriptional control to include signaling-related protein complexes.

### Quantitative Real- Time PCR (RT-qPCR) Analysis to Validate Predicted Genes

**Fig. 7:**
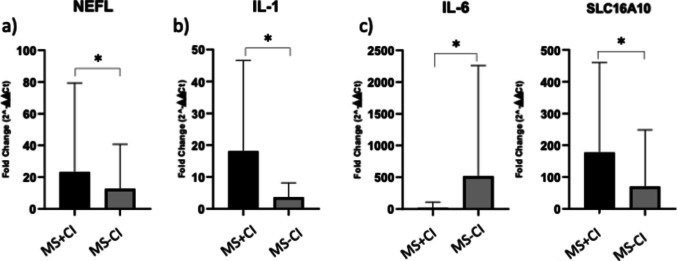
Column histograms displaying the expression levels in fold change of the five target genes on the *y*-axis and the sample of MS + CI and MS − CI labeled on the *x*-axis. Significance is marked as follows: *p-values from 0.05 to 0.01; **p-values between 0.01 and 0.001

Using the 2^−ΔΔCt^ approach, target genes (IL-1B, IL-6, SLC16A10, and NEFL) were consistently validated in samples of MS patients whose cognitive decline was evaluated through the neuropsychological BICAMS test. As shown in Fig. 7, NEFL, SLC16A10, IL-6, and IL-1B were statistically downregulated in MS-CI compared with MS + CI with corresponding *p*-values reported in Table 5.

**Table 5:** Target genes *p*-values in BICAMS group

Gene name	*p*-value < 0.05
IL-1B	0.0479231
IL-6	0.044764
SLC16A10	0.026574
NEFL	0.047579

### Correlation Analysis

The Pearson correlation coefficient (r) was used to establish a systematic correlation between the two variables (microRNAs and target genes), and the expression level data from RT-qPCR validation was used for this scope. For this analysis, only the microRNAs and target genes with a statistically significant difference in expression level between the two conditions, MS + CI and MS-CI, have been considered.

**Fig. 8:**
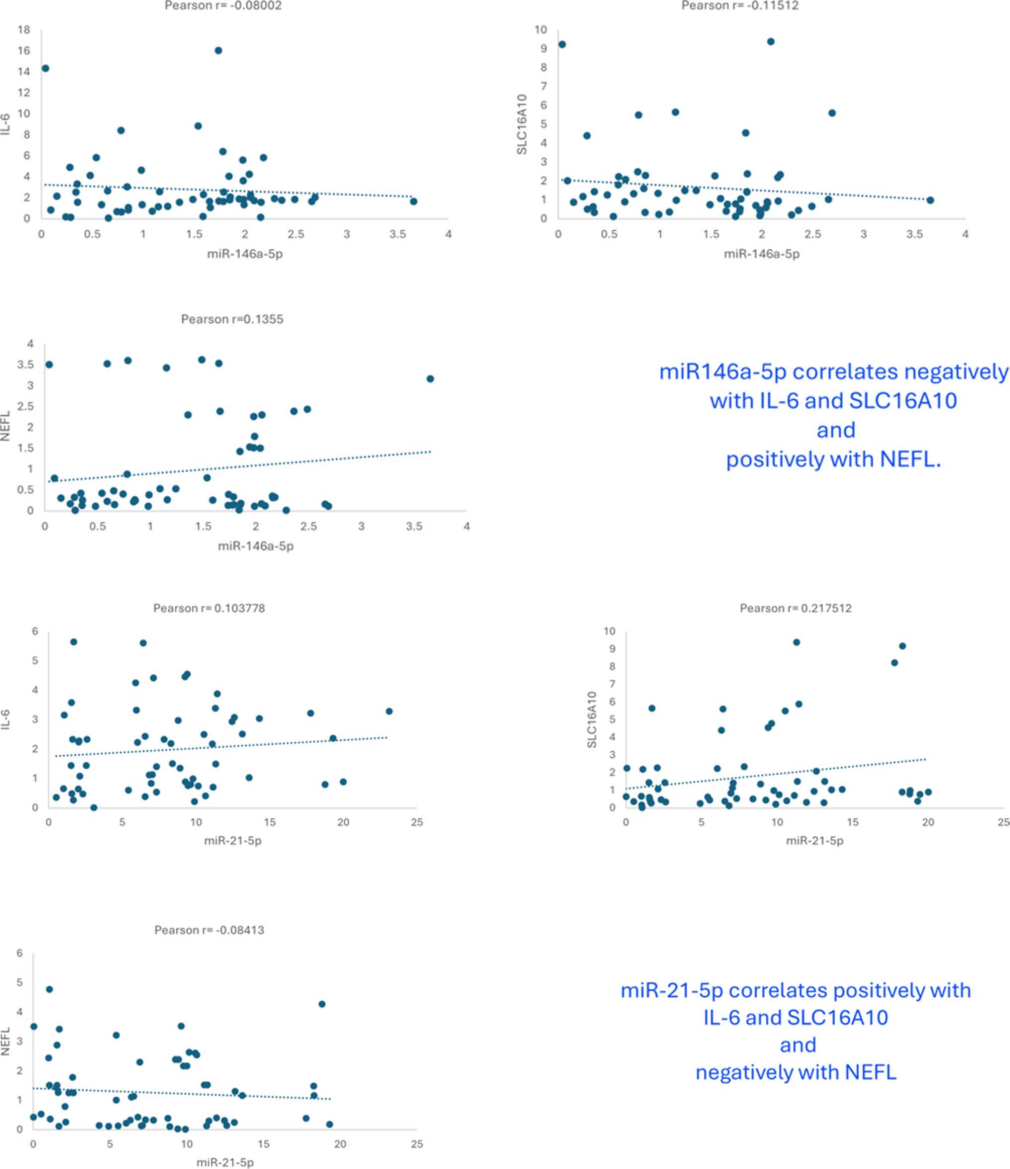
Scatter plot showing the Pearson correlation between miR-146a-5p and SLC16A10, IL-6 and NEFL (**A**); miR-21-5p and SLC16A10, IL-6 and NEFL (**B**)

Figure 8 shows the correlations that were statistically significant (*p* < 0.05). As illustrated in Fig. 8A, miR-146a-5p displayed a negative correlation with IL-6 and SLC16A10 expression and a positive correlation with NEFL. Figure 8B shows a positive correlation between miR-21-5p and SLC16A10 and IL-6 genes and a negative correlation with NEFL.

## Discussion

Over two million people worldwide suffer with multiple sclerosis (MS), an immune-mediated, inflammatory, demyelinating, and neurodegenerative disease of the central nervous system [33]. Cognitive impairment in MS is increasingly recognized as a clinically relevant manifestation that may occur even in patients with limited physical disability. In the present study, we identified a distinct peripheral miRNA–mRNA profile associated with cognitive impairment, characterized by reduced miR-146a-5p and let-7a-5p expression, increased miR-21-5p expression, and altered expression of inflammation- and neuroaxonal injury-related genes. Notably, these molecular differences were observed despite the absence of significant between-group differences in EDSS or conventional lesion burden, supporting the view that cognitive dysfunction in MS may reflect biological processes not fully captured by standard clinical-radiological measures.

The goal of our research was to find molecular markers associated with the BICAMS test results to support the diagnosis of cognitive decline in MS patients. MicroRNAs profiling identified miR-let-7a-5p, miR-146a-5p, and miR-21-5pas significantly dysregulated. RT-qPCR validation demonstrated a statistically significant down expression of miR-146a-5p and miR-let-7a-5p and overexpression and mir-21-5p in MS patients with cognitive decline compared to MS patients without cognitive decline. Target genes IL-1B, IL-6, SLC16A10, and NEFL were also validated concurrently. In MS patients with cognitive impairment, the four target genes chosen for analysis, as NEFL, IL-1 SLC16A10 and IL-6 exhibited statistically significant overexpression. This convergence is consistent with the view that cognitive dysfunction in MS may arise from the interaction of chronic immune activation, neurodegenerative processes, and altered regulatory control at the post-transcriptional level. The examination of all the data reveals that, even though all the samples were taken from MS patients, the MS patients with cognitive decline exhibit a different pattern of gene and microRNA expression compared to patients without cognitive decline.

MicroRNA-let-7a (miR-let-7a) targets the signal transducer and activator of transcription-3 (STAT3) and regulates microglia function [34] and activates the PI3K-Akt signaling pathway that promotes polarization of M2 macrophage, which is implicated in the modulation of inflammation and immunoreaction [35]. Although the role of miR-let-7a-5p in MS is not well understood, several scientific studies have found the low expression level of this miRNA in a variety of human diseases, especially in cancers. Zhang et al. [34] found that the expression of let-7a-5p was negatively correlated with TNF-α, IL-1B, and IL-6; again, let-7a-5p/IL-6 interaction regulates the inflammatory response through the Ras-MAPK pathway. It has been documented that the Ras-MAPK pathway is strongly linked to oxidative stress and inflammation, and that it regulates several distinct physiological processes, including transcription, apoptosis, differentiation, and proliferation of cells. IL-6 is a multidirectional, multifunctional cytokine that has significant physiological and pathological characteristics. It is involved in tissue damage, inflammation, and immunological response. Although direct evidence specifically linking let-7a-5p to cognitive impairment in adult MS remains limited, its known involvement in inflammatory signaling makes it biologically relevant in this context. In our network analysis, let-7a-5p showed broad regulatory connectivity and included IL6R among its validated targets, suggesting a potential contribution to cytokine-related pathways. Thus, rather than proposing a disease-specific role already established in MS, our data support let-7a-5p as a plausible component of a broader inflammatory-regulatory axis deserving further validation in cognitively characterized MS cohorts.

Among the dysregulated miRNAs, miR-146a-5p is particularly relevant in the context of MS because of its known role as a negative regulator of inflammatory signaling. Reduced miR-146a-5p expression has been associated with enhanced NF-κB-related activity and insufficient restraint of cytokine-mediated responses [36]. In our cohort, lower miR-146a-5p levels in MS + CI were associated with higher IL-6 and IL-1B expression, suggesting a reduced control of inflammatory signaling, contributing to the so-called smouldering inflammation that preferentially affects gray matter structures and synaptic networks critical for cognitive function. We observed higher NEFL expression in MS + CI. This persistent inflammatory state may contribute to cognitive dysfunction even in the absence of clear differences in focal lesion burden.

miR-21-5p showed the opposite pattern, being increased in cognitively impaired patients. This finding is in agreement with the broad literature describing miR-21 as a pro-inflammatory and immune-related miRNA in neurological and autoimmune disorders [37, 38]. In MS, miR-21 dysregulation has been reported in immune cells and has been associated with disease-related inflammatory activity [37, 38]. In the present study, its increase in MS + CI, together with the parallel upregulation of IL-6 and IL-1B, supports the presence of an activated inflammatory profile in cognitively impaired patients, strengthening the biological plausibility of a miRNA-associated inflammatory signature linked to cognitive status.

The mRNA validation data further supports the relevance of this profile. IL-1B and IL-6 are well-established mediators of neuroinflammation and immune activation in MS [39, 40]. Their increased expression in MS + CI is consistent with the idea that cognitive impairment may be associated with a more pronounced inflammatory state, even in the absence of clear differences in EDSS or conventional lesion measures. SLC16A10 encodes the aromatic amino acid transporter TAT1/MCT10. Thyroid hormones profoundly influence brain development and function, and variations in their levels can lead to neurological dysfunction, including memory impairment and altered mood [41]. This may point to altered metabolic and transport-related mechanisms and possibly indicate an imbalance between neuroprotective/anti-inflammatory and pro-inflammatory regulatory mechanisms in MS + CI [41–43], although its role in MS-related cognitive dysfunction remains to be clarified. NEFL is of particular interest because it is linked to neuroaxonal injury [15, 44, 45]. Its higher expression in MS + CI is consistent with the possibility that inflammatory dysregulation coexists with enhanced axonal stress in cognitively impaired patients.

The bioinformatic analyses support this integrated interpretation. The interaction network showed shared and interconnected targets across the three dysregulated miRNAs, while PPI and enrichment analyses pointed to transcriptional regulation, protein interaction networks, and inflammation-related signaling as major biological themes. Although these analyses do not directly demonstrate functional causality, they indicate that the identified miRNAs are part of a regulatory network plausibly linked to immune and neurodegenerative processes.

Our findings are also in line with previous reports suggesting that miRNA alterations may contribute to the biological substrate of cognitive dysfunction in MS. Recent literature has increasingly highlighted miRNAs as candidate molecular markers of disease heterogeneity and neurological impairment in MS [6, 19, 20], including studies specifically addressing cognitive dysfunction or related neuroinflammatory mechanisms [37, 38].

Correlation analysis was included as an exploratory step to assess directional associations between dysregulated miRNAs and selected target genes. Some miRNA–mRNA pairs reached nominal statistical significance; however, the observed correlation coefficients were modest. For this reason, these associations should be interpreted cautiously and not as evidence of direct regulatory effects in vivo. In particular, the stronger biological interpretation of the present study rests on the convergence between differential expression, target-network analysis, and the inflammatory/neuroaxonal relevance of the validated genes, rather than on the correlation analysis alone.

Our results are in line with previous evidence suggesting that miRNA dysregulation is involved in MS-related cognitive dysfunction. The integrative study by Prabahar and Raja highlighted miRNA-associated pathways relevant to MS cognition, including PI3K/Akt and ECM-related signaling, supporting the view that cognitive impairment reflects network-level molecular alterations [46]. In pediatric MS, Liguori et al. also found associations between circulating miRNAs and neuropsychological performance, together with cognition-related target-gene networks, although those findings were exploratory and did not survive multiple-testing correction [47]. Overall, despite differences in age, sample type, and analytic strategy, these studies support the plausibility of peripheral miRNAs as markers of cognitive dysfunction in MS. The same general direction is suggested by a study on miRNA dysregulation in MS-related cognitive impairment [48].

Taken together, our findings suggest a coordinated peripheral regulatory pattern rather than isolated expression changes. Reduced miR-146a-5p may weaken negative feedback on inflammatory signaling, favoring increased IL-6-related activity. At the same time, increased miR-21-5p is compatible with a pro-inflammatory immune profile and may reflect activation of peripheral immune cells relevant to MS pathobiology. The concomitant increase in NEFL expression supports the possibility that inflammatory dysregulation coexists with enhanced neuroaxonal stress or injury-related signaling. Within this framework, let-7a-5p may act as an additional regulatory layer influencing cytokine-associated pathways, including IL6R-linked signaling.

Some limitations should be acknowledged. First, the sample size was modest, and expression values showed considerable inter-individual variability. Second, the study lacks an independent replication cohort. Accordingly, the present results should be considered exploratory and hypothesis-generating.

Collectively, our data indicate that CI in MS is associated with a specific microRNA-gene regulatory profile reflecting the interplay between chronic inflammation, neuroaxonal damage, and metabolic dysregulation in MS. The combined pattern of reduced miR-146a-5p and let-7a-5p, increased miR-21-5p, and higher expression of IL-1B, IL-6, SLC16A10, and NEFL suggests an interconnected inflammatory-neuroaxonal axis associated with cognitive status. These findings provide a biologically plausible framework for future studies aimed at refining molecular stratification of cognitive dysfunction in MS.

## Conclusions

The cohort of MS patients, evaluated for their cognitive abilities, using the BICAMS test battery, was recruited to identify a possible correlation between a genetic component and the outcome of the BICAMS test. In this study, MS patients with cognitive impairment showed a distinct peripheral expression pattern characterized by reduced miR-146a-5p and let-7a-5p, increased miR-21-5p, and higher expression of IL-1B, IL-6, SLC16A10, and NEFL. Among the dysregulated miRNAs, the results most consistently supported a role for miR-146a-5p and miR-21-5p within an inflammatory regulatory framework, whereas let-7a-5p and correlations should be interpreted more cautiously as part of the broader dysregulated signature. These alterations were observed in groups that did not differ significantly in EDSS score or conventional lesion burden, suggesting that cognitive status may reflect biological processes that are not fully captured by routine clinical-radiological measures.

In conclusion, in BICAMS +, the panel composed of down expressed miR-146a-5p and let7a-5p, and overexpressed miR-21-5p, in association with overexpressed NEFL, IL-1 and SLC16A10 and IL-6, is an indicator of cognitive decline in MS disease and it could genetically validate the outcome of the BICAMS tests battery. This study identifies a distinct microRNA-gene expression profile associated with CI in patients with MS, independent of conventional clinical and radiological measures of disease severity, together with altered expression of their target genes involved in inflammation, neuroaxonal integrity, and metabolic processes, highlighting the contribution of epigenetic mechanisms to MS-related cognitive dysfunction. Importantly, the identification of a peripheral blood-based microRNA signature associated with CI opens new perspectives for the development of minimally invasive biomarkers for early detection, patient stratification, and monitoring of cognitive decline in MS. Additional mechanistic studies and eventual validation of these signatures could pave the way to personalized therapeutic strategies aimed at preserving cognitive function. Further investigation is required in this instance as well. Longitudinal studies in larger cohorts are warranted to validate the predictive and prognostic value of these molecular signatures and clarify their role in the temporal evolution of CI in MS. Overall, our data support the existence of a peripheral miRNA–mRNA profile associated with cognitive impairment in MS. These findings should be considered exploratory and hypothesis-generating, given the modest sample size, inter-individual variability, and lack of independent replication. Larger and longitudinal studies will be necessary to determine whether this molecular profile has predictive, stratification, or monitoring value in MS-related cognitive dysfunction.

## Data Availability

No datasets were generated or analysed during the current study.
